# In Response

**DOI:** 10.4269/ajtmh.2010.10-0114b

**Published:** 2010-07

Dear Sir:

We thank Van den Ende and others for their comment on our study, in which we assessed the utility of malaria RDTs in our specific population of HIV-positive adults with a low malaria prevalence which we attributed to cotrimoxazole prophylaxis and use of insecticide-treated bed nets. We concluded that “in similar populations of febrile outpatient HIV-positive adults, a negative Binax-M test can be useful for ‘ruling out’ malaria at the point of care.”

We agree with the authors of the letter that our findings do not apply to other settings or populations in which malaria prevalence is likely to be higher. Van den Ende and others suggest that the “diagnostic gain offered by the RDT” is low. However, in our setting, in the absence of an alternative test, presumptive treatment would generally be given to all suspected cases resulting in unnecessary malaria treatment and missed opportunities to detect and treat the actual cause of the febrile illness.

Although it is true that malaria is a life-threatening disease (especially in children), malaria is also an over-diagnosed and over-treated condition in resource-poor settings, especially within low-risk patient populations, such as ours. By the same token, fever in an HIV-infected person is life-threatening and complex to manage. Malaria RDTs have the potential to help rule out malaria in low-risk patients, facilitating empiric treatment and further diagnostic tests for the true cause of febrile illness.

Lisa A. Mills

Division of Infectious Diseases

Johns Hopkins School of Medicine

Baltimore, MD 21205

E-mail: lmills@ke.cdc.gov

Gertrude Nakigozi

Rakai Health Sciences Program

Kalisizo, Rakai District, Uganda

Ronald H. Gray

Johns Hopkins Bloomberg School of Public Health

Baltimore, MD 21205

Thomas C. Quinn

Steven J. Reynolds

National Institute of Allergy and Infectious Diseases

National Institutes of Health

Bethesda, MD 20892

